# Supplementary material for: Alcohol Contribution to Total Energy Intake and Its Association with Nutritional Status and Diet Quality in Eight Latina American Countries
Source: Int J Environ Res Public Health. 2021 Dec 13;18(24):13130. doi: 10.3390/ijerph182413130 (PMC8701082; doi:10.3390/ijerph182413130)
Supplement: Supplementary file 1 [file ijerph-18-13130-s001.zip › ijerph-1430777-supplementary.pdf]

Supplementary Table S1. Alcohol consumption, energy from alcohol and percentage of total energy intake according to socioeconomic characteristics among non-alcohol consumers.

		Alcohol consumption (g)				Energy from alcohol (Kcal)				Energy from alcohol (percentage of total energy intake)			
	N	mean	SE	Min	Max	mean	SE	Min	Max	mean	SE	Min	Max
Overall sample	8145	0.29	0.01	0.00	14.69	2.08	0.10	0.00	103.84	0.10	0.00	0.00	8.18
Sex													
Male	3676	0.32	0.02	0.00	14.44	2.24	0.16	0.00	102.12	0.10	0.01	0.00	7.26
Female	4469	0.27	0.02	0.00	14.69	1.94	0.14	0.00	103.84	7.26	0.01	0.00	8.18
Age group													
15–19 years	1164	0.19	0.03	0.00	14.13	1.38	0.22	0.00	99.89	0.06	0.01	0.00	5.76
20–34 years	3024	0.29	0.02	0.00	14.44	2.08	0.17	0.00	102.20	0.10	0.01	0.00	6.20
35–49 years	2291	0.3	0.03	0.00	14.69	2.13	0.19	0.00	103.84	0.11	0.01	0.00	8.18
50–65 years	1666	0.35	0.04	0.00	14.44	2.49	0.26	0.00	102.20	0.12	0.01	0.00	6.42
Countries													
Argentina	981	0.25	0.03	0.00	9.93	1.77	0.24	0.00	70.21	0.08	0.11	0.00	3.15
Brazil	1676	0.23	0.03	0.00	0.12	1.64	0.21	0.00	0.84	0.10	0.01	0.0	7.26
Chile	764	0.41	0.05	0.00	13.86	2.90	0.38	0.00	98.02	0.18	0.02	0.00	5.13
Colombia	1149	0.25	0.04	0.00	14.48	1.83	0.27	0.00	102.37	0.08	0.01	0.00	4.81
Costa Rica	731	0.23	0.04	0.00	13.65	1.60	0.30	0.00	96.51	0.08	0.02	0.00	4.68
Ecuador	767	0.45	0.05	0.00	14.44	3.15	0.37	0.00	102.12	0.13	0.02	0.00	5.76
Peru	1029	0.23	0.03	0.00	12.15	1.59	0.24	0.00	85.89	0.08	0.01	0.00	4.74
Venezuela	1048	0.39	0.06	0.00	14.69	2.76	0.39	0.00	103.84	0.12	0.02	0.00	8.18
Socioeconomic status													
Low	5014	0.31	0.02	0.00	14.69	2.20	0.14	0.00	103.84	0.11	0.01	0.00	8.18
Middle	3131	0.27	0.02	0.00	13.91	1.88	0.15	0.00	98.31	0.09	0.01	0.00	5.29
High													
